# Effects of Mixing Volatile Fatty Acids as Carbon Sources on *Rhodospirillum rubrum* Carbon Metabolism and Redox Balance Mechanisms

**DOI:** 10.3390/microorganisms9091996

**Published:** 2021-09-21

**Authors:** Paloma Cabecas Segura, Quentin De Meur, Audrey Tanghe, Rob Onderwater, Laurent Dewasme, Ruddy Wattiez, Baptiste Leroy

**Affiliations:** 1 Laboratory of Proteomics and Microbiology, University of Mons, 7000 Mons, Belgium; paloma.cabecassegura@umons.ac.be (P.C.S.); q.demeur@gmail.com (Q.D.M.); ruddy.wattiez@umons.ac.be (R.W.); 2 Materia Nova ASBL, Parc Initialis, Avenue Copernic 3, 7000 Mons, Belgium; Audrey.Tanghe@MATERIANOVA.BE (A.T.); Rob.Onderwater@MATERIANOVA.BE (R.O.); 3 Systems, Estimation, Control and Optimization Group, University of Mons, 7000 Mons, Belgium; laurent.dewasme@umons.ac.be

**Keywords:** purple bacteria, VFA, *Rs. rubrum*, dihydrogen, proteomic, redox homeostasis, photoheterotrophy

## Abstract

*Rhodospirillum rubrum* has a versatile metabolism, and as such can assimilate a broad range of carbon sources, including volatile fatty acids. These carbon sources are gaining increasing interest for biotechnological processes, since they reduce the production costs for numerous value-added compounds and contribute to the development of a more circular economy. Usually, studies characterizing carbon metabolism are performed by supplying a single carbon source; however, in both environmental and engineered conditions, cells would rather grow on mixtures of volatile fatty acids (VFAs) generated via anaerobic fermentation. In this study, we show that the use of a mixture of VFAs as carbon source appears to have a synergy effect on growth phenotype. In addition, while propionate and butyrate assimilation in *Rs. rubrum* is known to require an excess of bicarbonate in the culture medium, mixing them reduces the requirement for bicarbonate supplementation. The fixation of CO_2_ is one of the main electron sinks in purple bacteria; therefore, this observation suggests an adaptation of both metabolic pathways used for the assimilation of these VFAs and redox homeostasis mechanism. Based on proteomic data, modification of the propionate assimilation pathway seems to occur with a switch from a methylmalonyl-CoA intermediate to the methylcitrate cycle. Moreover, it seems that the presence of a mixture of VFAs switches electron sinking from CO_2_ fixation to H_2_ and isoleucine production.

## 1. Introduction

*Rhodospirillum rubrum* (*Rs. rubrum*), a purple non-sulfur bacteria (PNSB) belonging to the alpha-proteobacteria class, has been extensively studied for its versatile metabolism. This bacterium can assimilate a broad range of substrates, ranging from sugar to volatile fatty acids [1]. VFA_S_ very often result from waste stream treatment through fermentation [2], making them particularly interesting carbon sources for biotechnological processes [3,4,5,6,7]. The valorization of this type of carbon sources to produce high added value compounds such as pigments and biopolymers [2,8] using purple bacteria could help increase the profitability of the biobased industry and lead towards a circular economy. In waste streams, VFA_S_ are always found in mixtures. Even though the compositions and proportions of VFA_S_ depend on many factors, the most abundant VFA_S_ produced are usually acetate, butyrate, and propionate [9,10,11,12,13,14]. The metabolic process involved in the assimilation of these VFA_S_ has been studied in PNSB. In *Rs. rubrum,* for example, the net carbon assimilation of acetate occurs via the use of an alternative anaplerotic pathway, the ethylmalonyl-CoA pathway (EMC) [15,16,17]. When propionate is used as a carbon source, propionyl-CoA is carboxylated in methylmalonyl-CoA before being converted to succinate, which enters the tricarboxylic acid cycle (TCA) [18]. Concerning butyrate assimilation, a recent study carried out on *Rs. rubrum* highlighted the use of the EMC pathway for butyrate assimilation, as well as the newly proposed methylbutanoyl-CoA pathway (MBC) [19].

The previous studies were performed on culture supplemented with only one carbon source, meaning they did not properly reflect the events that occur under more environmental or engineered conditions, where several VFA_S_ would be simultaneously present. Some studies carried out on cultures of PNSB growing on a mixture of VFA_S_ already highlighted the synergic effect on the uptake rate of VFA_S_ and the sequential uptake of the various VFA_S_ simultaneously present in the medium [4,5]; however, the mechanisms involved in these phenomena have not yet been described. We recently showed that in presence of a mixture of VFA_S_, a lesser requirement for bicarbonate supplementation was observed in *Rs. rubrum* [20]. We also observed that propionate and acetate inhibited butyrate assimilation in binary mixtures [20]. In addition, we showed that when *Rs. rubrum* was cultivated using a mixture of propionate and butyrate, contrary to what is observed when these VFA_S_ are provided as sole carbon sources, bicarbonate supplementation was not required anymore. The bicarbonate supplementation is supposedly required to maintain the redox balance of the cell, with the fixation of CO_2_ being one of the main electron sinks in PSNB. This result suggests that alternative redox homeostasis mechanisms must be involved when a mixture of VFA_S_ is used as a carbon source. The present study aims, therefore, to unravel the way propionate affects butyrate assimilation, as well as the molecular aspects of propionate and butyrate assimilation when supplied as unique organic carbon sources or as a mixture in *Rs. rubrum.*

## 2. Materials and Methods

### 2.1. Bacterial Strain, Culture Medium, and Growth Conditions

The strains used was *Rhodospirillum rubrum* S1H (ATCC15903). The growth basal medium used for photoheterotrophic culture conditions of *Rs. rubrum* was the basal salt medium of Segers and Verstraete described by Suhaimi [21,22]. This basal medium was supplemented with NH_4_CI (35 mM) as the nitrogen source, NaHCO_3_ (3 mM or 50 mM), and biotin (0.06 mM). Different VFA_S_ were used as carbon sources, including a mixture of propionate (20.6 mM) and butyrate (15.5 mM), propionate (41.66 mM), or butyrate (31.25 mM). The pH was adjusted to 6.9. *Rs. rubrum* was grown under anaerobic phototrophic conditions in 50 mL sealed serum flasks under 50 µmole·m^−2^·sec^−1^ of light intensity supplied by halogen lamps (Sencys, Amsterdam, The Netherlands; 10 W; 100 lumens; 2650 K) at 30 °C with a rotary shaking at 200 rpm. Each culture condition was achieved with three biological replicates, except for the cultivation experiments with a mixture of acetate and excess butyrate, which were carried out on four biological replicates. Nitrogen gas was used to purge oxygen from the upper gas phase and the flasks were hermetically sealed. The cultures were inoculated at a starting OD_680nm_ range of 0.4–0.5 and the growth was monitored following the turbidity at OD_680nm._

### 2.2. Volatile Fatty Acid Consumption Monitoring by HPLC

The monitoring of VFA consumption by *Rs. rubrum* was performed on culture supernatants obtained by centrifugation at 16,000 g for 10 min at 4 °C and stored at −20 °C before analysis. Aliquots (100 µL) of the culture supernatant were analyzed by HPLC refractometry (Waters 2695 separation Module; Waters 2414 Refractive Index Detector, Waters, Milford, MA, USA). The separation was performed in isocratic mode using a Shodex Sugar SH1011 column (300 mm × 8 mm, SHODEX, New York, NY, USA) with 5 mM H_2_SO_4_ as the mobile phase. The detection was performed using refractometry at 210 nm. 

### 2.3. Polyhydroxyalkanoates Quantification

The method used to quantify polyhydroxyalkanoates content was adapted from a previously described method [23]. To determine the PHA composition and content, 500 µL of culture was centrifuged at 8000 rpm for 5 min. The pellet was resuspended with 500 µL of chloroform and added to a 10 mL screw cap glass tubes with 2 ml of a solution containing 1.94 mL of methanol, 0.06 mL of sulfuric acid, and 0.2 mg of toluic acid. The tubes were then placed in a thermostatically regulated bath at 100 °C for 210 min. After the reaction, 1 mL of distilled water was added and the tube was shaken vigorously. After phase separation, the organic phase (bottom layer) was removed and transferred to a small screw cap glass vial. The methyl esters were analyzed using gas chromatography using a SHIMADZU GC-MS QP2010S instrument (Shimadzu, Kyoto, Japan) equipped with an Optima ^®^ 5 capillary column (30 m/0.25 nm; Macherey-Nagels, Düren, Germany) and a flame ionization detector. Afterwards, 2 µL of the organic phase was analyzed after spitless injection. Helium (20.2 mL/min) was used as the carrier gas. The temperature of the injector was 250 °C.

### 2.4. Proteomic Analysis

Quantitative proteomic analysis was performed on protein extracts from *Rs. rubrum* S1H strain cultivated in butyrate, propionate, and a mix of propionate and butyrate containing medium with an excess of carbonate. Cells from 5 biological replicates were harvested by centrifugation (16,000× *g*, 10 min at 4 °C) when the OD_680nm_ reached 2.7. Proteins were extracted in a guanidine HCl 6M, K_2_HPO_4_ 50 mM buffer by sonication (3×10 s, amplitude 20%, IKA U50 sonicator). The protein concentration was determined using the Bradford method, with bovine gamma-globulin used as the standard [24]. Aliquots of extracted proteins (100 µg) were reduced using DTE, alkylated with iodoacetamide, and precipitated with acetone. The proteolytic peptides were obtained by overnight enzymatic digestion at 37 °C using trypsin at a ratio of 2:50 (*w/w*). Digestion was stopped with 0.1% formic acid (*v/v*, final concentration). The suspension was purified using Pierce™ C18 spin tips. The final amount of peptide was quantified using Pierce™ quantitative peptide. Proteins were identified and quantified following a label-free UHPLC-HRMS/MS platform (Eksigent Ekspert nano LC400 and Triple TOF™ 6600 AB Sciex, Singapore) in SWATH mode of acquisition. Peptides (2 µg) were separated on a C18 column (3 μm, 75 μm×15 cm, Dionex, Sunyvale, CA, USA) using a linear acetonitrile (ACN) gradient (2 to 35% of acetonitrile (*v/v*), 120 min) in water containing 0.1% (*v/v*) formic acid, at a flow rate of 300 nL/min^−1^. To achieve the greatest possible retention time stability, the column was equilibrated with 10 vol 5% acetonitrile (ACN) before each injection. Mass spectra were acquired over the range of 400–1250 *m/z* in high-resolution mode (resolution >35,000), with a 50 ms accumulation time. SWATH MS/MS spectra were acquired in data-independent acquisition (DIA) mode over the same *m/z* range with 50 variable SWATH windows. SWATH data were used to identify peptides via comparison with a reference spectral library obtained using a data-dependent acquisition (DDA) experiment on protein samples extracted from *Rs. rubrum* S1H grown on different carbon sources (succinate, acetate, propionate, butyrate, valerate, hexanoate) and with different light intensity levels (50 µmolm^−2^s^−1^, 150 µmolm^−2^s^−1^). The sample preparation and separation procedures used to construct the library were identical to those mentioned above. For library creation, the instrument was operated in data-independent acquisition mode and MS/MS spectra were acquired over the range of 100–1800 *m/z*. The precursor selection parameters were an intensity threshold of 200 c.p.s and accumulation time of 50 ms. Spectra were identified via comparison with the *Rs. rubrum* ATCC11170 UniProt entries (UPID/UP000001929/May 2013) using the AB Sciex ProteinPilot^TM^ 5.0 software. For peptide quantification, AB Sciex PeakView^TM^ 2.2 software and SWATH^TM^ processing were used to calculate XICs for the six highest fragments for the top five peptides from all identified proteins at a false discovery rate below 1%. Only unmodified and unshared peptides were subject to quantification. Peptides were also excluded when the confidence in their identification was below 0.99. A mass tolerance of 0.015 *m/z* and a retention time window of 2 min were also applied for XIC creation and integration. The areas under the curve of the XIC were exported to MarkerView TM 1.3. software for normalization based on the summed areas of the entire run and statistical analyses. Only proteins with fold changes, greater or lower than 1.5 or 0.66, *p*-values under 0.05 and quantified with at least two peptides were further considered. 

### 2.5. Isoleucine Quantification 

Branched amino acids (BCAAs) were extracted from pellets issued from the centrifugation of 500 µL cultures. The pellets were resuspended in 1.5 mL of methanol/chloroform solution (1:2, *v:v*). The resuspended pellets then underwent five freeze–thawing cycles, then 400µL of MilliQ water was added and the mixture was centrifuged (5000 rpm, 10 min, 4 °C). The upper aqueous phase was recovered and submitted to SpeedVac before being stored at −20 °C until analysis. The obtained pellet was then resuspended in mass spectrometer loading solvent consisting of 0.2% formic acid in ultrapure MS-grade water. The BCAA content was analyzed using an Eksigent LC425 system coupled to a Q-TRAP instrument (AB Sciex Q-Trap−6500+; ABSciex, Singapore) used in multiple reaction monitoring (MRM) modes. Peptides were separated on a C18 YMC-Triat 0.3 × 150 mm column operated at a flow rate of 5 µL/min in isocratic mode (3% acetonitrile (*v/v*), formic acid 0.1% (*v/v*)). The following transitions were used to quantify the following amino acids: arginine 175/116 and isoleucine 132/69. In order to avoid extraction bias, isoleucine abundance was expressed as the ratio of the area under the curve of the isoleucine transition to the area under the curve of the specific transition of the arginine.

### 2.6. H_2_ Quantification 

H_2_ was sampled from the culture headspace using a gas-tight syringe and was quantified using a Trace™ 1300 gas chromatograph (Thermofisher, Waltham, MA, USA) equipped with a thermal conductivity detector and a HayeSep N Sulfinert column (0.25 m by 1 nmm; Agilent, Santa Clara, CA, USA). Argon was used as the carrier gas at a flow rate of 1 mL/min. The oven temperature was 60 °C, the inlet temperature was 200 °C, and the detector temperature was 110 °C.

### 2.7. Acetolactate Synthase Specific Activity

Cells were harvested after 24 or 72 h of culture before being centrifuged and washed using phosphate buffer (50 mM, pH = 7.0). Cells were lysed in 100 µL phosphate buffer using 25 mg of glass beads and lysozyme (1 mg/mL final concentration), then the protein concentration was determined using the Bradford method [24], with bovine gamma globulin used as the standard. Acetolactate synthase activity was tested as described previously [25]. Acetolactate produced after one hour was assayed at a single endpoint by conversion to acetoin, which was detected using the reaction method described by Westerfeldt [26] and quantified through the use of a standard curve after subtraction of the acetoin produced without substrate. The acetoin content was then normalized based on the protein content. 

## 3. Results

**Figure 1:**
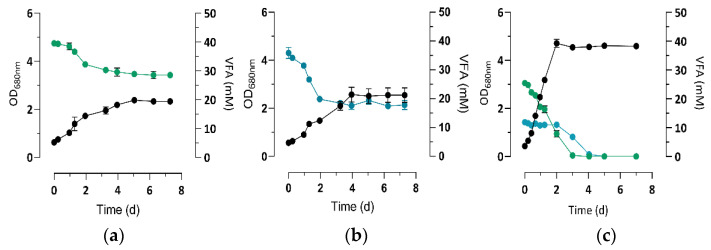
Monitoring of the growth (●) and VFA consumption in cultures of *Rs. rubrum* S1H cultivated with (**a**) propionate (●), (**b**) butyrate (●), or a mixture of both VFA_S_ (**c**), supplemented with 3 mM of bicarbonate. Data presented are averages of 5 biological replicates with standard deviations.

Based on the recently observed inhibition of acetate and propionate via the assimilation of butyrate in purple bacteria [20], we decided to study the molecular mechanism of propionate and butyrate assimilation by *Rs. rubrum* when supplied as a mixture. The VFA uptake profile showed that propionate induces a delay in the assimilation of butyrate, as previously observed in *Rs. rubrum* (Figure 1) [20]. The growth curve can consequently be divided into a propionate or a butyrate assimilation phase, as shown in Figure 1c.

**Figure 2:**
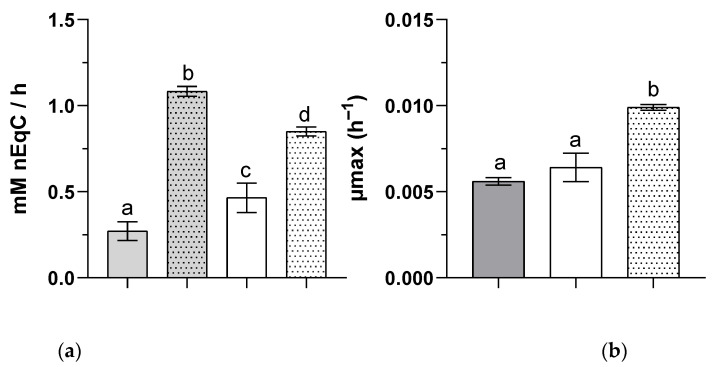
Carbon source consumption rate (**a**) and biomass growth rate (**b**) of *Rs. rubrum* grown using propionate (■), butyrate (□), or a mixture of these VFA_S_ (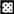) as the carbon source (*n* = 5). The letters a–d indicate statistically significant difference.

Surprisingly, while the supplementation of 3 mM of bicarbonate was not sufficient for complete assimilation of the 124 mM of net equivalent carbon coming from propionate or butyrate (Figure 1a,b), when they were used as sole carbon sources, they were totally consumed by the nominal 3 mM bicarbonate present in the culture medium when provided as a mixture of VFA_S_ (Figure 1c). This observation tended to indicate changes in the assimilation mechanism of propionate and butyrate when they are supplied together or individually. The presence of a cosubstrate also seems to induce faster growth of the biomass and faster removal of the carbon source (Figure 2), indicating a possible synergic effect due to the presence of several VFA_S_.

### 3.1. Proteomic Analysis of Rs. rubrum Cultivated on a Propionate and Butyrate Mixture 

To better understand the differences in propionate and butyrate assimilation when supplied separately or as a mixture, we compared the proteomes of the biomass obtained with butyrate and propionate as sole carbon sources, as well as under binary mixture conditions, at different phases of the growth curve, i.e., during the early propionate assimilation phase and the late butyrate assimilation phase (Figure S1). In order to ensure continuous and complete assimilation of butyrate and propionate when provided as sole organic carbon sources, culture mediums were supplemented with 50 mM of bicarbonate. To ensure proper comparison of the proteomic data obtained in the binary mixture and with butyrate and propionate only, the binary mixture was similarly performed with 50 mM bicarbonate supplementation. A total of 1891 proteins were identified and quantified (false discovery rate below 1%); these proteomic data covered about half of the theoretical proteome of *Rs. rubrum* S1 (ATCC1170, 3836 entries in Uniprot). Only proteins identified with more than 2 peptides and with significant (*p*-value < 0.05) and biologically relevant (<0.66 or >1.5) fold changes were further considered. The complete data set is available in the Supporting Materials (Table S1). 

### 3.2. Propionate and Butyrate Photoassimilation by Rs. rubrum When Present as the Sole Carbon Source

To clarify the impact of the presence of butyrate and propionate on their respective assimilation, it was necessary to first determine which proteins were specific to the assimilation of propionate or butyrate when supplied separately. We, thus, focused on proteins presenting a differential abundance between butyrate only and propionate only. Among the 277 proteins differentially regulated between these conditions, 135 presented fold changes lower than 0.66 (proteins more abundant under propionate condition) and 142 presented fold changes higher than 1.5 (proteins more abundant under butyrate conditions). The proteins discussed in the following paragraph are presented in Table 1. 

**Figure 3:**
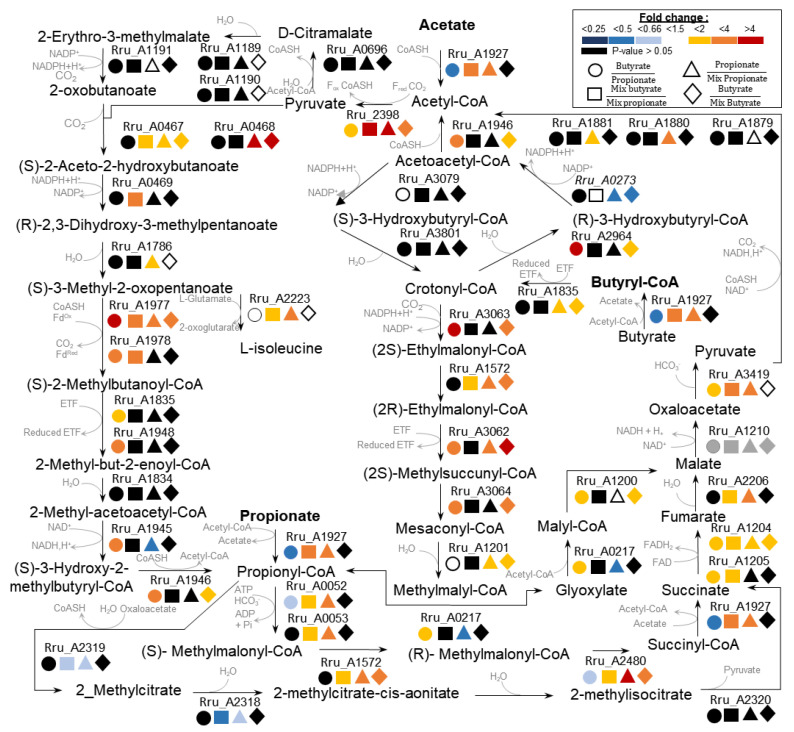
Schematic representation of the metabolic pathways highlighted through differential proteomic analysis (*n* = 5). The different colors indicate the fold changes: white, biologically non-relevant fold change (fold change value between 0.66 and 1.5); black, fold change not significant (*p*-value > 0.05); grey, protein not identified.

The data obtained were in favor of the assimilation of propionate through its conversion in succinyl-CoA via a methylmalony-CoA intermediate (Figure 3), confirming the previous reports [18]. This pathway requires five enzymes (Rru_A0052, Rru_A0053, Rru_A1572, Rru_A2480, Rru_A1927), among which three were significantly more abundant in propionate conditions than in butyrate conditions: the biotin carboxylase (Rru_A0052, fold change: 0.63), the methylmalonyl-CoA mutase (Rru_A2480, fold change: 0.61), and the succinyl-CoA transferase (Rru_A1927, fold change: 0.35). The carboxyl transferase, Rru_A0053, was also quantified in slightly higher abundance in propionate conditions (fold change: 0.83, *p*-value < 0.05), but did not reach the biological relevance threshold used in this study. Regarding proteins presenting a higher abundance in butyrate conditions, the data were consistent with a previous proteomic analysis carried out in our lab, showing assimilation of butyrate through both the EMC and MBC pathways (Figure 3) [19]. Indeed, the butyryl-CoA dehydrogenase (Rru_A1835, fold change: 1.97) involved in the conversion of butyryl-CoA to crotonyl-CoA was upregulated under butyrate conditions, as was the cluster of key enzymes involved in the EMC pathway (Rru_A3062, fold change: 2.62; Rru_A3063, fold change: 6.17; Rru_3064, fold change: 2.23) (Table 1).

A higher abundance of proteins involved in the newly proposed MBC pathway, which combined branched-chain amino acid biosynthesis and degradation, was also noticed. The biosynthesis of BCAA responsible for the conversion of acetyl-CoA into (S)-3-methyl-2-oxopentanoate was not significantly upregulated under butyrate conditions; however, the indole oxidoreductase (Rru_A1977 and Rru_A1978) that connect the BCAA biosynthesis pathway to the BCAA degradation pathway was more abundant under butyrate conditions than propionate conditions (Rru_A1977, fold change: 4.86; Rru_A1978, fold change: 3.32), with the same observation being made for the cluster of enzymes involved in the isoleucine degradation pathway (Rru_A1945, fold change: 2.17; Rru_A1946, fold change: 2.63; Rru_A1948, fold change: 3.14) (Table 1). These results support the previous hypothesis that propionate is assimilated through the methylmalonyl-CoA pathway, while butyrate is assimilated through the EMC and MBC pathways when these carbon sources are present separately.

### 3.3. Photoassimilation of Butyrate and Propionate by Rs. rubrum When Present as a Mixture

#### 3.3.1. Methylcitrate Cycle

In contrast to previous observations, the number of enzymes involved in the conversion of propionyl-CoA in succinyl-CoA via the methylmalonyl-CoA intermediate pathway was significantly higher during the butyrate assimilation phase (Table 1). Comparing the abundances of these enzymes between propionate-only conditions and the propionate assimilation phase under binary mixture conditions also revealed a higher abundance when propionate was the only carbon source (Table S1). These observations suggested a lower dependency of propionate assimilation on the methylmalonyl-CoA pathway when butyrate was simultaneously present in the medium. On the other hand, the citrate synthase (Rru_A2319) and the 2-methylcitrate dehydratase (Rru_A2318) were more abundant during the propionate assimilation phase under binary mixture conditions (Rru_A2318, fold change: 0.47; Rru_A2319, fold change: 0.53) (Figure 3). The 2,3-dimethylmalate lyase (Rru_2320) also presented a higher abundance during the propionate assimilation phase (fold change: 0.66), although this fold change was not statistically significant (*p*-value: 0.34). The citrate synthase and 2-methylcitrate dehydratase were also more abundant when propionate was assimilated in a binary mixture than when this VFA was provided as a sole carbon source (Rru_A2318, fold change: 0.61; Rru_A2319, fold change: 0.52). These enzymes are involved in the methylcitrate cycle, a metabolic pathway involved the assimilation of propionate [27]. The data, thus, indicate a potential change in the metabolic pathway used for propionate assimilation when butyrate is present in the medium, with a higher dependency on the methylcitrate cycle.

#### 3.3.2. EMC and MBC Pathways

Under butyrate only conditions, proteomic data indicated that both EMC- and MBC-pathway-related enzymes were more abundant, as mentioned above. Interestingly, under binary mixtures condition, only enzymes of the MBC pathway were still more abundant during the butyrate assimilation phase. Indeed, the cluster of key enzymes of the EMC pathway (Rru_A3062, Rru_A3063, Rru_A3064) was no longer upregulated during the assimilation of butyrate (Table 1). Comparing butyrate only conditions with the butyrate assimilation phase under binary mixture conditions, these three enzymes were observed to be significantly more abundant under the butyrate only conditions (Rru_A3062, fold change: 4.48; Rru_A3063, fold change: 3.12; Rru_A3064, fold change: 2.41) (Table S1). These data suggested a lower dependency of butyrate assimilation on the EMC pathway when *Rs. rubrum* was grown on a binary mixture. On the other hand, some of the enzymes involved in the first part of the MBC pathway and corresponding to the BCAA biosynthesis pathway (acetolactate synthase large subunit (Rru_A0467), pyruvate-flavodoxin oxidoreductase, NiFJ (Rru_A2398), and ketol acid reductoisomerase (Rru_A0469)) were observed in higher abundance during the butyrate assimilation phase under binary mixture conditions. On the other hand, these enzymes were systematically more abundant when butyrate and propionate were present separately than during their respective assimilation phase in the binary mixture. We recently suggested that in *Rs. Rubrum,* transient isoleucine accumulation could be an electron sinking mechanism, with its synthesis involved in consumption of the reducing equivalent [19,28].

**Figure 4:**
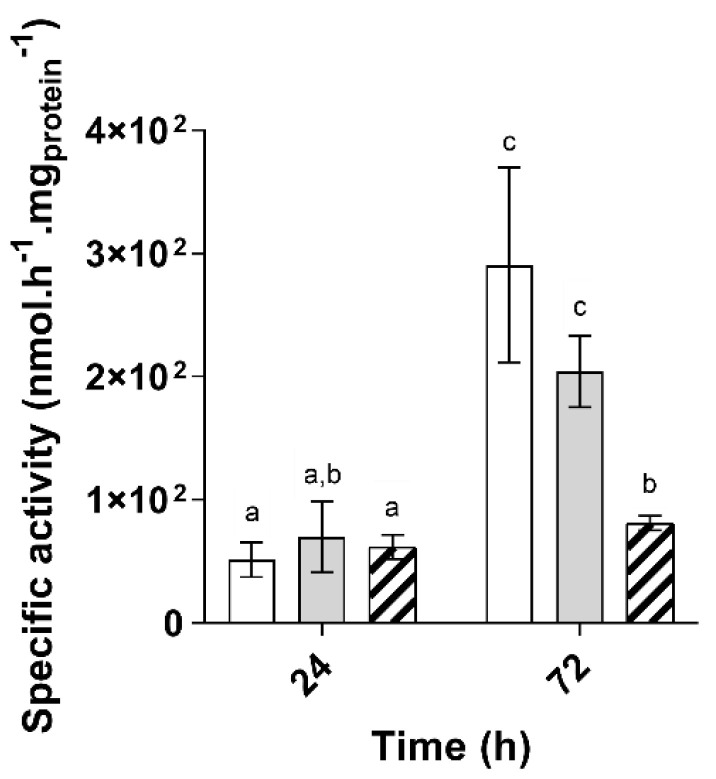
Differential expression of the enzymatic activity of *Rs. rubrum* acetolactate synthase in culture using propionate (■), butyrate (□), or a mixture of VFA_S_ in culture supplemented with 50 mM of bicarbonate (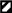) (*n* = 5). The letters a–c indicate statistically significant difference.

Altogether, these observations suggest that the BCAA synthesis pathway is more active when propionate and butyrate are present separately rather than in a mixture and potentially that more isoleucine is produced under these conditions as well. To test this hypothesis, we monitored the activity of the acetolactate synthase in cells obtained in butyrate and propionate only conditions and from the binary mixture after 24 and 72 h of cultivation. The acetolactate activity per mg of total proteins was significantly higher in samples obtained at 72 than 24 h (Figure 4) in all three conditions and significantly higher at 72 h in propionate and butyrate only conditions than in the binary mixture. Since proteomic samples from propionate and butyrate only conditions were collected at 56 and 48 h, respectively (Figure S1), the results of this activity test agree with the proteomic data. The potential activity of the BCAA synthesis pathway is effectively increased at later stages of the exponential growth phase, but also in propionate and butyrate only conditions as compared to the binary mixture after 72 h of culture. 

#### 3.3.3. Redox Homeostasis in *Rs. rubrum* When Butyrate and Propionate Are Used as Sole Organic Carbon Sources or as a Mixture

During photoheterotrophic growth, the oxidative pathways converting reduced organic substrates into biosynthetic precursors produced ample reducing power. To maintain the central metabolic flow, a large amount of the produced reducing equivalent must be recycled through an electron sinking pathway. Several redox balancing reactions have been proposed to fulfil this function, potentially including the synthesis of isoleucine described previously [28]. 

Carbon dioxide fixation is probably the most common reaction used by PNSB to maintain the pool of oxidized electron carriers. The Calvin–Benson–Bassham cycle (CBB) has been extensively described as the key pathway for carbon dioxide fixation through the activity of Rubisco (Rru_A2400) [29]. Rubisco was highly upregulated under butyrate only conditions in comparison with propionate only conditions (fold change: 4.20); however, the upregulation was less important (fold change: 2.68) and not significant (*p*-value: 0.18) between the butyrate assimilation phase and the propionate assimilation phase under binary mixture conditions. In addition, while there was no significant change in the abundance of Rubisco under butyrate only conditions in comparison with the butyrate assimilation phase under binary mixture conditions (*p*-value: 0.27), the enzyme was more abundant during the assimilation phase of propionate under binary mixture conditions in comparison with the propionate only conditions (Fold change: 0.36) (Table S1). It seems that the presence of butyrate, alone or in the binary mixture, systematically triggered an increased abundance of Rubisco, even during the propionate assimilation phase. Nonetheless, the activity of Rubisco and the CBB cycle is linked with the consumption of bicarbonate ions from the culture medium or CO_2_ produced by the catabolic reaction, with the former only participating in electron sinking. The lesser requirement for bicarbonate supplementation observed in binary mixtures suggested that alternative electron sinking mechanisms are activated in the presence of multiple VFA_S_ and that Rubisco upregulation in this context could be linked with a higher requirement for catabolic CO_2_ capture. 

Other electron sinking pathways have been described in *Rs. Rubrum,* such as the reductive tricarboxylic acid cycle (rTCA) [30,31]. One of the key enzymes in this pathway, which is indicative of a functional reductive TCA, is the 2-oxoglutarate oxidoreductase (Rru_A2721, Rru_A2722). The 2-oxoglutarate oxidoreductase β subunit (Rru_A2722) was significantly more abundant under mixture conditions during both propionate (fold change: 0.46) and butyrate (fold change: 0.22) assimilation phases than under butyrate or propionate only conditions; however, the abundance of the 2-oxoglutarate oxidoreductase α subunit (Rru_A2721) was not significantly different in any condition (Table S1). A functional rTCA cycle could be an additional electron sinking pathway allowing the cell to maintain its redox homeostasis; however, further experiments will be needed to confirm the activity of the rTCA under mixture conditions.

**Figure 5:**
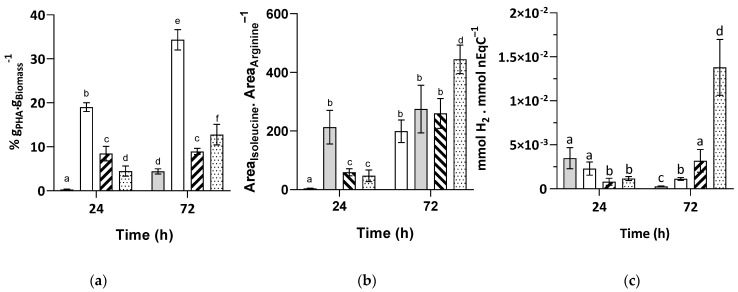
Differential accumulation of PHA (**a**), isoleucine (**b**), and dihydrogen (**c**) in *Rs. rubrum* culture using propionate (■), butyrate (□), or a mixture of these VFA_S_ as carbon sources supplemented with 50 mM of bicarbonate (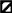) or 3 mM of bicarbonate (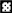) (*n* = 5). The letters a–f indicate statistically significant difference.

PHA production is very often proposed to also act as an electron sink [32]. We recently showed that this biopolymer accumulated in cells upon a sudden increase in light intensity, potentially in response to redox imbalance [33]. Proteomic data showed that PHA synthase (Rru_A2413) was highly upregulated under butyrate only conditions as compared to propionate only or binary mixture conditions and was invariant during the different assimilation phases in the binary mixture. To evaluate whether PHA also followed the same trend, we analyzed the PHA contents in cells obtained in all three conditions at 24 and 72 h. As expected from the proteomic data, *Rs. rubrum* accumulated the largest amount of PHA under butyrate only conditions, both at 24 and 72 h (Figure 5a). In the binary mixture, on the other hand, the PHA content remained relatively low during the entire culture phase. This observation was similar to the data obtained for the additional mixture of VFA_S_ containing acetate, propionate, butyrate, and valerate, whereby the mixture always showed lower levels of PHA than under single organic carbon source conditions [20]. To determine whether low PHA accumulation in a binary mixture can be linked with the supplementation of the culture medium with 50 mM of bicarbonate, we measured the PHA accumulation rates in cells grown in a binary mixture of propionate and butyrate supplemented with only 3 mM of bicarbonate. When a slight increase was observed, the level of accumulation of PHA was still much lower than under butyrate only or acetate only conditions [33]. Consequently, we concluded that PHA was not involved in the electron sinking mechanism that allowed *Rs. rubrum* to assimilate propionate and butyrate when provided as a binary mixture without bicarbonate assimilation.

As we proposed that isoleucine accumulation could be linked with redox balancing and to test whether *Rs. rubrum* effectively accumulated isoleucine in the studied conditions, we monitored the intracellular isoleucine contents in all three conditions at 24 and 72 h. The isoleucine content, as determined using LC-MS/MS and normalized based on the arginine content, was observed to be higher under butyrate conditions at 24 h, confirming our previous observation [19] (Figure 5b). At 72 h, the isoleucine content was also increased under propionate conditions, as expected from the proteomic analysis and acetolactate activity tests. Unexpectedly, an increase in the isoleucine content was observed in the binary mixture after 72 h of growth. No upregulation of the enzymes of the threonine-dependent isoleucine synthesis pathway was observed under binary mixture conditions, although isoleucine could be produced by this alternative pathway. 

The accumulation of isoleucine could potentially be explained by the decreased abundance during the butyrate assimilation phase under mixture conditions in comparison with butyrate only conditions of the 2-oxoacid dehydrogenase family protein Rru_A1977, which is supposedly involved in linking isoleucine biosynthesis and degradation in the MBC assimilation pathway. It could be hypothesized that a lowered abundance of this pivotal enzyme of the isoleucine degradation, even without upregulation of the isoleucine synthesis pathway under binary mixture conditions, could explain the isoleucine accumulation under mixture conditions. When only 3 mM of bicarbonate was provided, an increase in the isoleucine/arginine ratio was observed after 72 h of culture, suggesting that the isoleucine synthesis pathway is involved in redox homeostasis when propionate and butyrate are simultaneously use as carbon sources. 

In some strains constitutively expressing nitrogenase, the production of H_2_ was shown to replace the CBB cycle as the electron sink [34]. The production of H_2_ was, therefore, quantified to see whether this redox balance mechanism occurred under mixture conditions. No major significant differences were observed when the culture was supplemented with 50 mM of bicarbonate (Figure 5c); however, in a culture grown with a mixture of propionate and butyrate as the carbon source and supplemented with only 3 mM of bicarbonate, a significant increase in H_2_ production was observed. The amount obtained was lower than the results found in the literature when malate was used as a carbon source [34]; however, this increase in H_2_ production when the availability of bicarbonate decreased could explain the lower requirement for bicarbonate supplementation under mixture conditions. Using our proteomic data set, it was not possible to determine the metabolic pathway involved in this process. Indeed, no changes in the abundance of proteins potentially involved in the production of H_2_ (hydrogenases, nitrogenase, or CO-dehydrogenase) were observed in our proteomic analysis (Table S2). It has to be noted that as the carbon nitrogen ratio at the beginning of the culture was 3.54, the production of H_2_ likely occurred under nitrogen non-limiting conditions, which is highly unusual. Further analysis would be necessary to understand the synthesis of H_2_ in propionate–butyrate mixtures and to determine whether or not this phenomenon could be observed in other mixtures. 

## 4. Discussion

### 4.1. Mixing VFA_S_ Leads to Synergy in Their Assimilation

The presence of several carbon sources appears to induce a synergic effect, as the highest µ_max_ was observed under mixed VFA_S_ conditions. A similar synergic effect was reported for photosynthetic cultures of a consortium of purple bacteria, whereby combinations of acetate with butyrate or propionate increased the uptake rates of VFA_S_ [4]. This phenomenon could result from the fact that with such mixtures, the biosynthesis of some precursors might be facilitated. For example, the balance of acetyl-CoA synthesis from butyryl-CoA is 0.5 Butyryl-CoA + 0.5 ETF_oxy_ + 0.5 NADP^+ →^ acetyl-CoA + 0.5 ETF_red_ + 0.5 NADPH,H^+^; whereas from propionyl-CoA, the balance is propionyl-CoA + GDP+ Pi+ FAD^+^ + 2NAD^+^ + H_2_O ^→^ GTP + 2 NADPH,H^+^ + FADH_2_ + CO_2_ + acetyl-CoA. Our proteomic analysis highlighted the use of the methylcitrate cycle for the assimilation of propionate in the presence of butyrate, whereas under propionate only conditions the methylmalonyl-CoA pathway seemed to be used. Referring to the pyruvate production, while the balance of the methylcitrate cycle is 1 propionyl-CoA + 2 H_2_O + FAD^+^ + NAD^+^ + ATP ^→^ 1 Pyruvate + FADH_2_ + NADH + H^+^, the balance of the production of pyruvate via the methylmalonyl-CoA is 1 propionyl-CoA + FAD^+^ + NAD^+^ H_2_O ^→^ CoASH + FADH_2_ + NADH + H^+^ + 1 pyruvate. Based on the balance of each pathway, they seem to be equivalent regarding the CO_2_ or reducing power consumption; however, the difference between these assimilation routes could be due to the lower energetic cost of the methylcitrate cycle and the recovery of the energy needed to activate propionate by the transfer of the CoASH. In the methylmalonyl-CoA pathway, during the conversion of succinyl-CoA in succinate, the CoASH is supposedly transferred to acetate to form acetyl-CoA, while during the conversion of propionyl-CoA in methylcitrate, the CoASH seems to be hydrolyzed and is not recovered. This difference might make the methylcitrate pathway less efficient at this level. On the other hand, producing acetyl-CoA can be achieved more easily from butyryl-CoA than through methylmalonyl-CoA when propionate and butyrate are mixed, explaining the faster growth under mixed conditions. Another possible explain for the use of the methylcitrate cycle under mixture conditions could be the lower concentration of propionate. A previous study on *Burkholderia sp.* showed a change in the assimilation pathway used for propionate assimilation between a methylcitrate intermediate and the acrylate pathway according to the level of propionate in the medium [35]. At a low level, propionate was assimilated via the methylcitrate pathway, while at a high level its assimilation involved the acrylate pathway [35,36]. A similar phenomenon could occur under mixture conditions when the level of propionate is lower than under propionate-only conditions.

### 4.2. Mixing VFAs Abolishes the Need for Bicarbonate Supplementation for the Assimilation of Reduced Substrates

In the absence of oxygen or other electron acceptors, oxidative phosphorylation cannot recycle all of the reduced cofactors produced during oxidative reactions; hence, the recycling of cofactors must occur via alternative pathways, described as electron sinks. Several redox balancing reactions have been proposed, although carbon dioxide fixation is probably the most common reaction used to maintain the pool of oxidized electron carriers and is strongly related to the redox state of the carbon source; the more reduced the carbon source, the higher the requirement for CO_2_ fixation, potentially requiring exogenous CO_2_ supplementation [37]. Surprisingly, while the supplementation of the culture medium with bicarbonate is a requirement observed with the most reduced carbon sources, such as propionate [38], butyrate [38], and to a lesser extent acetate [16,39], we recently showed that this supplementation is no longer required if *Rs. rubrum* is photoheterotrophically grown with mixtures of those three VFAs [20]. It could be hypothesized that the initial amount of bicarbonate available in the culture medium (3 mM) might allow the complete assimilation of the lower amounts of propionate and butyrate provided as a mixture in this study; however, experiments carried out on butyrate and propionate used as sole carbon sources showed that the nominal 3 mM of bicarbonate present in the mixture allowed the assimilation of less than 10 mM of propionate or 15 mM of butyrate, while the initial 20.6 mM and 15.5 mM of propionate and butyrate, respectively, were entirely assimilated when used as a mixed carbon source [20].

The lower requirement of bicarbonate supplementation could also be due to a change in the metabolic pathway involved in the assimilation of propionate and butyrate when they were simultaneously present [35,36]. For instance, the assimilation of butyrate in a binary mixture with propionate seemed to preferentially occur through the MBC pathway rather than the EMC pathway, with the latter not being particularly upregulated under these growth conditions. The EMC pathway consumes CO_2_, meaning carbon assimilation metabolism based on pathways that are less dependent on carbon dioxide, such as the MBC pathway, might be consistent with the observed lower need for bicarbonate supplementation under mixture conditions. Moreover, a kinetic proteomic analysis performed in butyrate showed that the abundances of most of the enzymes involved in the EMC pathway were relatively constant during the whole growth phase [19]. The absence of activation of this pathway is not likely to be related to an apparent “end of grow phase” phenotype but rather to the presence of propionate in the medium. Combining VFAs, therefore, seems to definitely impact the way they are assimilated. 

### 4.3. Mixing VFAs Changes the Mechanism Involved in the Redox Balance of the Cells

As dependency on bicarbonate fixation is decreased under mixtures of propionate and butyrate, and as CO_2_ fixation is known to be one of the main redox balancing mechanisms in purple bacteria, one may ask how redox balancing occurs under such conditions. Alternative electron sinking mechanisms may be used, such as reverse TCA or PHA production. 

One of the characteristic enzymes of the rTCA cycle, the 2-oxoglutarate oxidoreductase, was identified in the proteomic data set and quantified as having a higher abundance under mixture conditions compared to conditions involving either VFA as a single carbon source; however, although these may be the first results, the presence of this enzyme is not sufficient by itself and further analysis will be needed to support the hypothesis of an operational rTCA cycle during assimilation of a mixture of butyrate and propionate.

In *Rs. rubrum* growing on VFA, the production of PHA has been described as a possible redox balance mechanism. The production of hydroxybutyrate or hydroxyvalerate from propionate results in consumption of the redox equivalent; however, when production occurs from butyrate, PHA synthesis cannot play the role of an electron sink. It was hypothesized that under butyrate conditions, the PHA production can be used as a buffer zone to regulate the production of the reduced equivalent, making PHA synthesis a possible redox balance mechanism [19,40]. We recently linked the presence of propionate to a low level of PHA accumulation [20]. The presence of propionate in our mixture could, thus, potentially explain the lower PHA production observed under such conditions. The low PHA production rate in our binary mixture indicated that PHA production was not used here as a major electron sinking reaction and could not counterbalance the observed lower bicarbonate requirement. 

Studies carried out on *Rs. rubrum* grown on VFAs have highlighted a possible involvement of branched-chain amino acid accumulation, in particular isoleucine, as a possible electron sink during *Rs. rubrum* growth on acetate and butyrate [19,28,41]. In a mutant culture lacking Rubisco, the addition of α-ketoglutarate-derived amino acids inhibited the activity of the BCAA synthesis pathway and prevented the bacterial grow unless DMSO was added, indicating the involvement of this pathway in the redox balance maintenance of the cells [41]. Moreover, it was shown that in a culture of *Rs. rubrum* subjected to a rapid increase in light intensity, a phenomenon known to create redox stress [33], an increase in the isoleucine/arginine ratio occurred [28]. This indicated the importance of the BCAA synthesis pathway as a redox balance mechanism during the growth of *Rs. rubrum* under photoheterotrophic conditions. In culture supplemented with a mixture of propionate and butyrate and an initially low amount of carbonate (3 mM), the measured level of isoleucine was significatively higher than that observed under butyrate conditions, indicating that isoleucine production could be involved in the bicarbonate-independent redox balancing observed under mixed condition. 

Finally, the focus was shifted to H_2_ production, as it was demonstrated that H_2_ synthesis could replace the CBB cycle as an electron sink in strains of *Rs. rubrum* constitutively expressing nitrogenase [29,34]. Under mixture conditions, a significant increase in H_2_ production was observed when cultures were grown with a mixture of propionate and butyrate as the carbon source, especially when the medium was supplemented with only 3 mM of bicarbonate. The ratio observed between the quantity of H_2_ produced per net equivalent of carbon assimilated was lower than the value found in the literature for this strain, especially when propionate or butyrate was used as the sole carbon source [34]; however, the presence of a nitrogen source in the medium could explain why H_2_ was only produced in small quantities. Further analysis would be required to better understand this increase in H_2_ production, especially in terms of the mechanism triggering this synthesis under nitrogen non-limiting conditions.

## 5. Conclusions

The use of mixed VFAs appears to have a synergistic effect on growth phenotypes. Moreover, it seems that the presence of a mixture of VFAs removes the requirement for carbonate supplementation to allow the assimilation of butyrate and propionate by triggering H_2_ production and isoleucine accumulation. The concomitant use of these diverse electron sinking pathways could be a first step toward understanding the reduced need for external electron acceptors under mixture conditions; however, the reason why these mechanisms are inefficient when only one VFA is used as a carbon source still requires further investigation. The results obtained in this study open the door to a better understanding and more efficient utilization of PNSB in biotech processes using VFAs.

## Figures and Tables

**Table 1:** Differential expression of proteins in *Rhodospirillum rubrum* cultivate with propionate, butyrate, or a mixture of both. * The fold change is the ratio of the abundance of a protein under butyrate conditions to the abundance under propionate conditions. ** The fold change corresponds to the ratio of the abundance of a protein during the assimilation phase of butyrate and its abundance during the propionate assimilation phase under mixture conditions. *** No. of identified peptides represents the number of peptides used for quantification.

Uniprot Accession Number	Locus Tag	Fold Change (Butyrate/Propioanate) *	P-Value	Fold Change (Mix Butyrate/Mix Propionate) **	P-Value	No. of Identified Peptides ***	Description
Methylmalonyl-CoA pathway							
tr|Q2RYD8	Rru_A0052	0.63	3.41 × 10^−2^	1.81	9.50 × 10^−3^	5	Biotin carboxylase
tr|Q2RYD7	Rru_A0053	0.83	8.70 × 10^−2^	1.81	4.95 × 10^−2^	6	Carboxyl transferase
tr|Q2RU23	Rru_A1572	1.30	3.49 × 10^−1^	1.67	1.00 × 10^−2^	5	Methylmalonyl-CoA epimerase
tr|Q2RRG6	Rru_A2479	0.72	8.21 × 10^−2^	1.75	9.56 × 10^−2^	6	Methylmalonyl-CoA mutase
tr|Q2RRG5	Rru_A2480	0.61	3.43 × 10^−2^	1.56	1.37 × 10^−3^	6	Methylmalonyl-CoA mutase
Methilcitrate cycle							
tr|Q2RRX7	Rru_A2318	0.76	4.60 × 10^−1^	0.47	1.22 × 10^−2^	4	2-methylcitrate dehydratase
tr|Q2RRX6	Rru_A2319	0.25	1.61 × 10^−1^	0.53	3.96 × 10^−2^	5	Citrate synthase
tr|Q2RRX5	Rru_A2320	1.07	8.75 × 10^−1^	0.66	3.42 × 10^−1^	2	2.3-dimethylmalate lyase
Ethylmalonyl-CoA pathway							
tr|Q2RXX3	Rru_A0217	1.97	1.38 × 10^−2^	0.72	4.46 × 10^−1^	6	Citrate lyase
tr|Q2RXR7	Rru_A0273	0.556	1.00 × 10^−1^	0.74	7.70 × 10^−3^	5	3-oxoacyl-eductase
tr|Q2RV43	Rru_A1201	1.48	8.73 × 10^−4^	1.34	3.48 × 10^−1^	6	MaoC-like dehydratase
tr|Q2RTB0	Rru_A1835	1.97	1.43 × 10^−2^	1.49	1.41 × 10^−1^	4	Butyryl-CoA dehydrogenase
tr|Q2RT18	Rru_A1927	0.35	1.60 × 10^−2^	2.33	9.63 × 10^−3^	6	Acetyl-CoA hydrolase
tr|Q2RPS1	Rru_A3079	1.49	7.00 × 10^−3^	1.05	3.24 × 10^−1^	6	3-hydroxyacyl-CoA dehydrogenase
tr|Q2RQ36	Rru_A2964	1.03	8.16 × 10^−1^	0.87	3.28 × 10^−1^	2	MaoC-like dehydratase
tr|Q2RPT8	Rru_A3062	2.62	2.59 × 10^−2^	1.59	1.68 × 10^−1^	5	Methylmalonyl-CoA mutase
tr|Q2RPT7	Rru_A3063	6.17	4.78 × 10^−2^	0.76	2.34 × 10^−1^	6	Crotonyl-CoA reductase
tr|Q2RPT6	Rru_A3064	2.23	4.96 × 10^−2^	1.22	5.03 × 10^−1^	6	Isovaleryl-CoA dehydrogenase
tr|Q2RSZ9	Rru_A1946	2.63	5.35 × 10^−3^	1.18	3.40 × 10^−1^	5	Acetyl-CoA C-acetyltransferase
tr|Q2RMQ0	Rru_A3801	1.05	5.90 × 10^−1^	0.93	6.26 × 10^−1^	6	Short chain enoyl-CoA hydratase
Methylbutanoyl-CoA pathway							
tr|Q2RX73	Rru_A0467	1.25	3.69 × 10^−1^	1.66	1.51 × 10^−2^	6	Acetolactate synthase. large subunit
tr|Q2RX72	Rru_A0468	0.99	8.96 × 10^−1^	1.05	7.30 × 10^−1^	3	Acetolactate synthase. small subunit
sp|Q2RX71	Rru_A0469	1.58	6.27 × 10^−2^	2.02	4.40 × 10^−3^	6	Ketol-acid reductoisomerase
tr|Q2RX33	Rru_A0508	1.40	4.91 × 10^−1^	1.07	9.34 × 10^−1^	1	Aminotransferase
tr|Q2RWJ5	Rru_A0696	0.47	9.37 × 10^−2^	1.10	8.65 × 10^−1^	1	RNA methyltransferase
sp|Q2RV55	Rru_A1189	0.92	4.49 × 10^−1^	1.12	8.59 × 10^−2^	5	3-isopropylmalate dehydratase large subunit
sp|Q2RV54	Rru_A1190	1.06	5.53 × 10^−1^	1.49	2.54 × 10^−1^	6	3-isopropylmalate dehydratase small subunit
sp|Q2RV53	Rru_A1191	0.83	8.25 × 10^−2^	0.87	1.96 × 10^−1^	6	3-isopropylmalate dehydrogenase
sp|Q2RTF9	Rru_A1786	0.92	4.73 × 10^−1^	1.04	6.44 × 10^−1^	6	Dihydroxy-acid dehydratase
tr|Q2RTB1	Rru_A1834	1.16	4.20 × 10^−1^	1.26	9.54 × 10^−2^	2	Enoyl-CoA hydratase/isomerase
tr|Q2RT00	Rru_A1945	2.17	4.24 × 10^−3^	1.11	5.24 × 10^−1^	5	Short-chain dehydrogenase/reductase
tr|Q2RSZ7	Rru_A1948	3.14	3.47 × 10^−2^	0.91	7.65 × 10^−1^	2	Isovaleryl-CoA dehydrogenase
tr|Q2RSW8	Rru_A1977	4.86	3.21 × 10^−2^	3.13	1.81 × 10^−2^	2	Pyruvate ferredoxin/flavodoxin oxidoreductase
tr|Q2RSW7	Rru_A1978	3.32	2.41 × 10^−2^	2.02	4.90 × 10^−2^	6	Indolepyruvate ferredoxin oxidoreductase
tr|Q2RS72	Rru_A2223	0.79	2.10 × 10^−1^	1.87	4.90 × 10^−2^	6	2-keto-4-methylthiobutyrate aminotransferase
sp|Q53046	Rru_A2398	1.79	4.72 × 10^−2^	11.31	4.22 × 10^−2^	6	Pyruvate-flavodoxin oxidoreductase
Tricarboxylic carbon cycle							
tr|Q2RV44	Rru_A1200	1.87	2.59 × 10^−3^	1.53	1.06 × 10^−1^	5	Citrate lyase
tr|Q2RV40	Rru_A1204	1.82	1.58 × 10^−2^	1.55	4.84 × 10^−3^	6	MaoC-like dehydratase
tr|Q2RV39	Rru_A1205	1.98	3.64 × 10^−2^	1.54	4.01 × 10^−2^	5	MaoC-like dehydratase
tr|Q2RT66	Rru_A1879	0.82	2.98 × 10^−1^	1.03	7.28 × 10^−1^	6	Dihydrolipoamide acetyltransferase
tr|Q2RT65	Rru_A1880	0.85	3.22 × 10^−1^	1.08	3.59 × 10^−1^	6	Pyruvate dehydrogenase beta subunit
tr|Q2RT64	Rru_A1881	1.00	9.92 × 10^−1^	0.98	9.21 × 10^−1^	4	Pyruvate dehydrogenase
tr|Q2RS89	Rru_A2206	0.79	1.46 × 10^−1^	1.72	1.14 × 10^−3^	6	Fumarase
tr|Q2RNT2	Rru_A3419	1.88	6.88 × 10^−3^	3.02	1.35 × 10^−3^	6	Phosphoenolpyruvate carboxykinase
Calvin-Benson-Bassham cycle							
sp|Q2RRP5	Rru_A2400	4.20	3.45 × 10^−2^	2.68	1.86 × 10^−1^	6	Ribulose bisphosphate carboxylase
Reverse Tricarboxylic carbon cycle							
tr|Q2RQS7	Rru_A2721	0.96	9.26 × 10^−1^	1.52	1.48 × 10^−1^	2	2-oxoglutarate synthase. alpha subunit
tr|Q2RQS6	Rru_A2722	0.99	9.75 × 10^−1^	1.50	2.75 × 10^−2^	5	2-oxoglutarate synthase, beta subunit

## Data Availability

Not applicable.

## Supplementary Materials

The following are available online at https://www.mdpi.com/article/10.3390/microorganisms9091996/s1: Figure S1: Monitoring of the growth and butyrate and propionate consumption rates in a culture of *Rs. rubrum* S1H cultivated in propionate, butyrate, or a mixture of both, along with the proteomic sample times. Table S1: Full data set for the proteomic analysis carried out on *Rs. Rubrum*. Table S2: Differential expression of enzyme possibly involved in the H2 production.
