# Supplementary material for: Effects of Mixing Volatile Fatty Acids as Carbon Sources on Rhodospirillum rubrum Carbon Metabolism and Redox Balance Mechanisms
Source: Microorganisms. 2021 Sep 21;9(9):1996. doi: 10.3390/microorganisms9091996 (PMC8471276; doi:10.3390/microorganisms9091996)
Supplement: Supplementary file 1 [file microorganisms-09-01996-s001.zip › Figure S1.pdf]

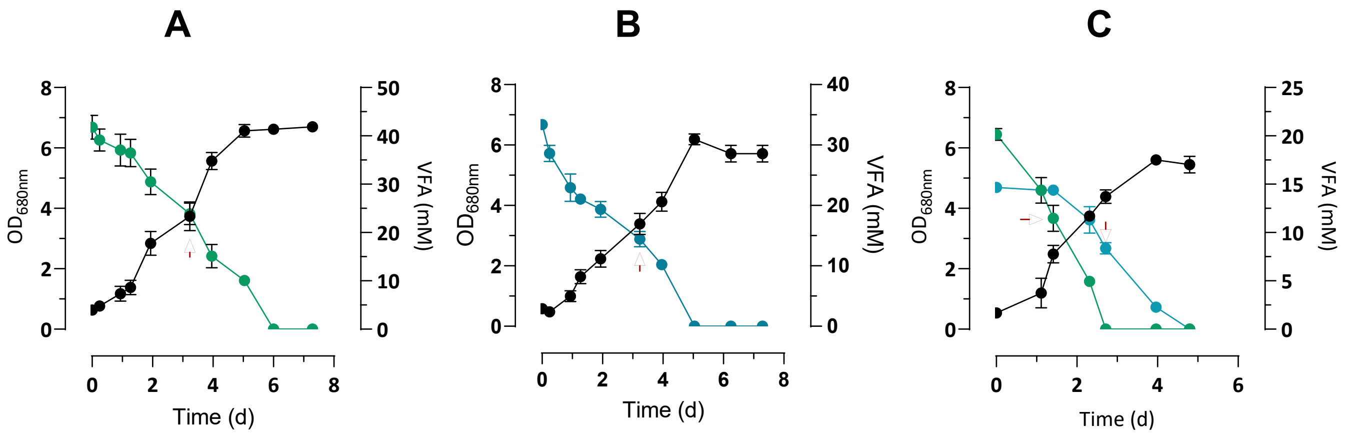

Figure S1 : Monitoring of the growth(●) and butyrate (●) and propionate (●) consumption in a culture of *Rs. rubrum* S1H cultivated in propionate (A), butyrate (B) or a mixture of both (C). The growth (opened markers; n=5) of culture realized with a mixture of VFAs was monitored by OD_{680nm}. The red arrows correspond to sample taken for proteomic analysis.
